# Hypocotyl Elongation Inhibition of Melatonin Is Involved in Repressing Brassinosteroid Biosynthesis in *Arabidopsis*

**DOI:** 10.3389/fpls.2019.01082

**Published:** 2019-09-26

**Authors:** Fangjie Xiong, Fengping Zhuo, Russel J. Reiter, Lingling Wang, Zhenzhen Wei, Kexuan Deng, Yun Song, Ghulam Qanmber, Li Feng, Zuoren Yang, Fuguang Li, Maozhi Ren

**Affiliations:** ^1^School of Life Sciences, Chongqing University, Chongqing, China; ^2^School of Chemistry and Chemical Engineering, Chongqing University of Science and Technology, Chongqing, China; ^3^Department of Cellular and Structural Biology, UT Health San Antonio, San Antonio, TX, United States; ^4^Zhengzhou Research Base, State Key Laboratory of Cotton Biology, Zhengzhou University, Zhengzhou, China; ^5^State Key Laboratory of Cotton Biology, Institute of Cotton Research, Chinese Academy of Agricultural Sciences, Anyang, China

**Keywords:** melatonin, hypocotyl inhibition, BR biosynthesis, gene expression, Arabidopsis

## Abstract

Melatonin functions as a plant hormone/regulator in the regulation of growth and development. However, the underlying mechanisms are still unclear. In this study, we found that a high dose of melatonin inhibited hypocotyl elongation in a dose-dependent manner in *Arabidopsis*. An expression profile analysis showed that hypocotyl growth inhibition by melatonin was involved in reprograming the expression of cell elongation genes and brassinosteroid (BRs) biosynthetic genes. Furthermore, similar to BR biosynthetic inhibitor brassinazole (BRZ), a high concentration of melatonin upregulated BR-biosynthetic genes and downregulated BR-induced genes involved in cell elongation, while melatonin was inefficient in brassinazole-resistant mutants like the *bzr1-1D* and *bes1-D* in hypocotyl inhibition. The comparative expression profile analysis showed an opposite expression mode in the co-regulated genes between melatonin and BZR1 or melatonin and brassinolide (BL). Additionally, exogenous BL rescued the repressive phenotype of BR biosynthesis-deficient mutant like *det2-1* even in the presence of high-dose melatonin, but not BR receptor mutant *bri1-5* or signal transduction mutant *bin2-1*. A biochemical analysis further confirmed that melatonin reduced endogenous BR levels in a dose-dependent manner in *Arabidopsis.* Taken together, these results indicate that melatonin inhibits BR biosynthesis but does not block BR signaling in the inhibition of hypocotyl elongation and extends insights on the role of melatonin in cross-talking with plant hormone signaling.

## Introduction

Melatonin, chemically *N*-acetyl-5-methoxytryptamine, is a low-molecular-weight substance found in living organisms ranging from bacteria to animals, with pleiotropic biological activities (Hardeland et al., 2006; Hardeland et al., 2011; Reiter et al., 2017). In plants, melatonin has a wide range of physiological functions such as regulating plant stress tolerance, senescence retardation, and pathogen defense. Additionally, melatonin also participates in the processes of root growth and architecture, ﬂowering, seed germination, and fruit ripening (Arnao and Hernández-Ruiz, 2014; Arnao and Hernández-Ruiz, 2015; Wei et al., 2015; Nawaz et al., 2016; Kanwar et al., 2018). Regarding growth regulation, melatonin has been found to promote growth in shoots, roots, and also explants. Melatonin reportedly stimulated root generation and cotyledon expansion, induced new lateral and adventitious roots, and also extended the coleoptiles in some monocot plants (Murch et al., 2001; Hernandez-Ruiz et al., 2005; Pelagio-Flores et al., 2013; Sarropoulou et al., 2012; Hernandez et al., 2015; Erland et al., 2018). Melatonin also acts as a growth promoter inducing active growth of hypocotyls in etiolated seedlings of lupin at micromolar concentrations while displaying an inhibitory effect at 100 μM (Hernandez-Ruiz et al., 2004). Generally, growth inhibition by melatonin only occurs at a high concentration (> 100 μM). High-concentration melatonin repressed root growth and reduced biomass, as well as retarded plant leaf growth by reducing both cell size and cell proliferation (Wang et al., 2017; Lin et al., 2018). Although melatonin plays an important role in the regulation of plant growth and development, the underlying mechanism is still unclear and remains to be elucidated.

In early studies, melatonin was considered primarily as an auxin-like effector in regulating of plant growth. However, to date, the accumulated data suggested that melatonin functions as a new plant hormone or regulator with versatile and complex activities in which it participates in the regulation of gene expression related to plant hormones including auxin, cytokinin, gibberellins (GAs), abscisic acid, ethylene, jasmonic acid, and salicylic acid (Arnao and Hernández-Ruiz, 2018; Arnao and Hernández-Ruiz, 2019). Melatonin showed effects on inducing expression of phytohormone-related genes, such as biosynthesis, catabolism, receptors, and transcription factors, and also influencing the endogenous plant hormone levels and their physiological actions. A recent investigation showed that a drop in melatonin by knockdown of the key enzyme gene like the *SNAT2* (serotonin *N*-acetyltransferase 2) and *TDC* (tryptophan decarboxylase), involved in melatonin biosynthesis, resulted in downregulation of the expression of *DWARF4* gene and reduction on endogenous brassinosteroid (BR) levels as well as a semi-dwarf phenotype in rice (Hwang and Back, 2018; Lee and Back, 2018), implying that there exists a close relationship between melatonin and the BR signaling pathway.

BRs, a class of plant-specific steroid hormones, play vital roles in regulating plant cell growth and morphogenesis, particularly in hypocotyl cell elongation, as the BR biosynthetic mutants, such as *de-etiolated 2* (*det2*), are defective in hypocotyl elongation and shows a dwarf phenotype (Li et al., 1996; Fujioka and Sakurai 1997; Clouse and Sasse, 1998). In BR signaling, BRs are perceived by the BR receptor BR-insensitive 1 (BRI1), which initiates a signaling cascade to inactivate the negative regulator BR insensitive 2 (BIN2), thereby activating the downstream transcription factors brassinazole-resistant 1 (BZR1) and *bri1*-EMS suppressor 1 (BES1), two key transcription factors mediating BR responses (Li and Chory, 1997; Li and Nam, 2002; Xu et al., 2008; Wang et al., 2011; Wang et al., 2012; Zhu et al., 2013). BZR1 and BES1 play central roles in BR-regulated gene expression and plant development. Activated BZR1 and BES1 accumulate in the nucleus and promote stem elongation through the transcriptional activation of their target genes, such as *PER1*, *IAA19*, *small auxin upregulated RNA* (*SAUR*), and *Expansin A8* (*EXPA8*), involved in cell elongation (Sun et al., 2010; Yu et al., 2011; Bai et al., 2012; Wang et al., 2012, Nolan et al., 2017), whereas negatively regulated by feedback suppression of expression of BR-biosynthetic genes like *DWF4*, *CPD*, and *BR6OX2* (Mathur et al., 1998; Asami et al., 2001; Wang et al., 2002; Fujioka and Yokota, 2003; He et al., 2005).

In this study, we found that melatonin inhibited hypocotyl elongation and reprogramed expression of cell elongation genes and BR-biosynthetic genes in a dose-dependent manner in *Arabidopsis*. Through a transcriptome analysis in combination with gene expression analysis, we also found that melatonin inhibited hypocotyl elongation involved in repressing BR pathway. Additionally, we demonstrated that melatonin inhibited BR biosynthesis but did not block BR signaling in the regulation of hypocotyl elongation in *Arabidopsis*. Our findings provide new insights into the function of melatonin in the regulation of plant hormone signaling pathways.

## Materials and Methods

### Plant Materials and Growth Conditions

The wild-type (WT) *Arabidopsis* Columbia (Col-0) ecotype was used unless otherwise indicated. The mutants used in this study include BR-related mutants *det2-1* (Chory et al., 1991), *bri1-5* (Noguchi et al., 1999), *bin2-1* (Peng et al., 2008), *bzr1-1D* (Wang et al., 2002), and *bes1-D* (Yin et al., 2002), in which *bri1-5 and bes1-D* are in Wassilewskija (Ws) and Ertkheim-2 (En2) backgrounds, respectively, and the others are in Col-0 background. Seeds were surface sterilized with 70% (v/v) ethanol for 2 min, followed by 10% (v/v) bleach solution for 5 min, and rinsed with sterile water five times. The seeds were then placed on 1/2 strength Murashige and Skoog (MS) medium (pH = 5.8) containing 1% sucrose and 0.8% agar. Seeds on medium were grown in growth chambers with 22°C and 16-h/8-h light/dark cycle settings unless indicated otherwise.

### Hormone or Inhibitor Treatments and Hypocotyl Elongation Assays

As for the observation of hypocotyl experiments, seeds were incubated 1/2MS medium with various concentrations of melatonin (MEL; Sigma, M5250) or 0.1% dimethyl sulfoxide (DMSO; solvent control) for 7 days or more in the light conditions. For another treatment, 4-day-old seedlings were transferred on medium plates supplemented with melatonin, brassinazole (BRZ; Sigma, SML1406) dissolved in DMSO, brassinolide (BL; Sigma, B1439), indole acetic acid (IAA; Sigma, I5148), gibberellin (GA_3_, Sigma, G7645), or the combination for treatment in darkness for another 3 days. BL, IAA, and GA_3_ were dissolved in 80% ethanol. After the indicated time of incubation, about 40 seedlings were laid horizontally on an agar plate and photographed. Hypocotyl length was measured using the ImageJ software (http://imagej.nih.gov/ij/) from digitally computed images of seedlings.

### RNA-Seq Analysis

RNA-Sequencing was carried out by BGI Life Tech Co. Ltd. (Wuhan, China). Four-day-old seedlings were transferred on 1/2MS plate containing 1 mM of melatonin or 0.1% DMSO (control). After 3 days of incubation in a dark condition, about 100 seedlings were collected as one replicate, and a total of three replicates were used for each treatment. Total RNA was isolated using RNeasy Plant Mini Kit (Tiangen, China) following the manufacturer’s protocol. The cDNA libraries were constructed, and the library quality was determined by using Bioanalyzer 2100 (Agilent), subsequently sequenced using the BGISEQ-500 platform (BGI). Clean reads obtained from the raw reads were obtained by removing the adapter and low-quality sequences and then mapped to the annotated genome sequence of *Arabidopsis* in The Arabidopsis Information Resource (TAIR) database (https://www.arabidopsis.org/) by using HISAT and Bowtie2 software (Langmead and Salzberg, 2012; Kim et al., 2015). Transcript abundance was also normalized by transforming the data to reads per kilobase of exon model per million mapped reads (FPKM) method, and DEGseq method was used to identify differentially expressed genes (DEGs) (Wang et al., 2010). A twofold difference in expression levels with an adjusted *p*-value ≤ 0.001 (*q*-value ≤ 0.001) was set as the threshold for the determination of the significant changes (Storey and Tibshirani, 2003). Gene ontology (GO) enrichment and Kyoto Encyclopedia of Genes and Genomes (KEGG) pathway analysis were carried out with the web tool DAVID bioinformatics resources 6.8 (http://david.abcc.ncifcrf.gov/home.jsp) (Huang da et al., 2009a; Huang da et al., 2009b) and KOBAS (http://kobas.cbi.pku.edu.cn/home.do) (Kanehisa et al., 2008; Kanehisa et al., 2016). All RNA-Seq data are available in **Supplementary Table S1**.

### Comparative Analysis of RNA-Seq Data

RNA-Seq was compared with the public online data from published articles. DEGs with 1.5-fold difference were set to keep the consistence between the data. Venn diagrams for expression differences were generated online (http://bioinfogp.cnb.csic.es/tools/venny/). Expression levels of overlapping DEGs were exhibited by heatmaps created by using the MeV4.9.0 software (Saeed et al., 2003). The overlapping DEGs are listed in **Supplementary Table S2–S3**.

### RNA Isolation and Real-Time PCR (qRT-PCR) Analysis

Total RNA was isolated by using the RNAprep Pure Plant Kit (TianGen, DP432), and reverse transcription was performed using the PrimeScript™ RT Reagent Kit (Takara, RR047A) following the manufacturer’s instructions. Real-time polymerase chain reaction (qRT-PCR) was performed on a Bio-Rad CFX96 System (Bio-Rad) using the TB Green™ Premix Ex Taq™ (Takara, RR420A). *ACTIN2* was used as internal control for BR-related experiments. Primer sequences used for qRT-PCR analyses are listed in **Supplementary Table S4**.

### Bioactive BRs Determination by ELISA

Ten-day-old seedlings grown on 1/2MS, including various concentrations of melatonin (100, 500, and 1,000 μM), 1 μM of BRZ, or 0.1% DMSO (control), were collected for determining BR levels. Bioactive BR contents were quantified by enzyme-linked immunosorbent assay (ELISA) as described previously (Hwang and Back, 2018).

### Data Processing

The presented results were obtained from three independent experiments. Data were expressed as means ± standard deviations (SDs) of 40 plants (*n* ≥ 40) with each measurement performed in triplicate. The data were evaluated by a one-way analysis of variance (ANOVA), followed by Student tests at a signiﬁcance level of *p* < 0.05 (*) or extremely significant level of *p* < 0.01 (**).

### Accession Numbers

Information for the main genes involving in this study is available in The TAIR (http://www.arabidopsis.org/) under the following accession numbers: *Paclobutrazol resistance1* (*PRE1*) (AT5G39860), *IAA19* (AT3G15540), *ACS5* (AT5G65800), *SAUR15* (AT4G38850), *SAUR16* (AT4G38860), *EXPA8* (AT2G40610), *DWF4* (AT3G50660), *CPD* (AT5G05690), *BR6OX2* (AT3G30180), and *ACTIN2* (AT3G18780).

## Results

### Melatonin Inhibits Hypocotyl Elongation of *Arabidopsis*

**Figure 1:**
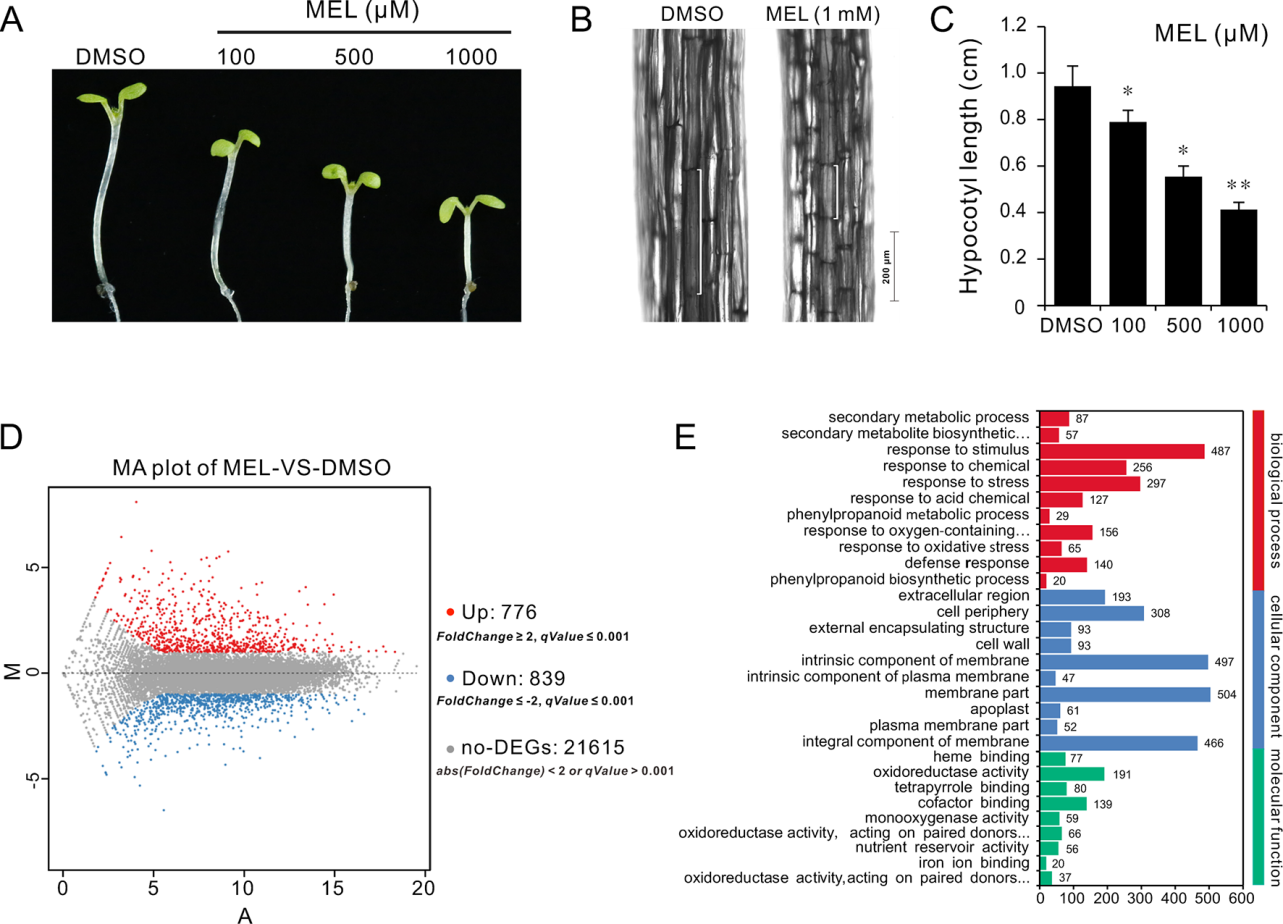
RNA-Seq of analysis genes affected by melatonin. Seedlings of four DAGs were transferred on 1/2MS plate containing different concentrations of melatonin (100, 500, and 1,000 µM) or 0.1% DMSO (solvent control). After 3 days of incubation in a dark condition, seedlings from 1 mM of melatonin and DMSO treatment were used for gene expression profile analysis by RNA-Seq. **(A,** **B)** Melatonin inhibits hypocotyl and cell elongation in a dose-dependent manner. **(C)** Hypocotyl length of seedlings. Error bars indicate the SEM (*n* ≥ 40). *p < 0.05, **p < 0.01 (Student’s *t* test). **(D)** DEGs affected by 1 mM of melatonin. **(E)** The top 30 of the most enriched GO terms (*q*-value < 0.01). DAG, days after germination; DMSO, dimethyl sulfoxide; GO, gene ontology; MS, Murashige and Skoog; SEM, standard error of the mean.

Melatonin as a plant growth regulator promotes or inhibits plant growth depending on the concentration of melatonin. To understand how melatonin affects plant growth, a series of concentrations of melatonin (0, 1, 10, 100, 500, and 1,000 μM) were set to assess its effects on seedling growth. After 10 days of germination, coincident with previous report, seedlings grown on 1/2MS plates with high-dose melatonin showed obvious growth arrest with smaller cotyledon and true leaves compared with those in solvent control (**Supplementary Figure S1A**). Melatonin reportedly promotes hypocotyl growth in etiolated lupin (*Lupinus albus*) at a micromolar concentration level, while it has an inhibitory effect at high concentrations (Hernandez-Ruiz et al., 2004). Similarly, our observation showed that the *Arabidopsis* seedlings also displayed a repressed hypocotyl and cell length by high-dose melatonin in a dose-dependent manner (**Supplementary Figures S1B, C**). Specifically, seedlings had displayed slight response to low-dose melatonin (1 and 10 μM); however, when concentrations of melatonin were increased (100, 500, and 1,000 μM), seedlings showed considerable reduction in hypocotyl length, which declined to about 80%, 60%, and 40%, respectively, compared with that of solvent control (**Supplementary Figure S1D**). To extend these findings and confirm inhibition in cell elongation of high dose of melatonin, we transferred 4 days seedlings to 1/2MS plates with various concentrations of melatonin (100, 500, and 1,000 μM) and incubation in a dark condition to better observe cell elongation. After 3 days of incubation, similar results were observed in which seedlings showed obvious reduction in hypocotyl and cell length as with increasing dose of melatonin in comparison with solvent control (**Figures 1A–C**).

### Melatonin Reprograms Cell Elongation Gene Expression

**Figure 2:**
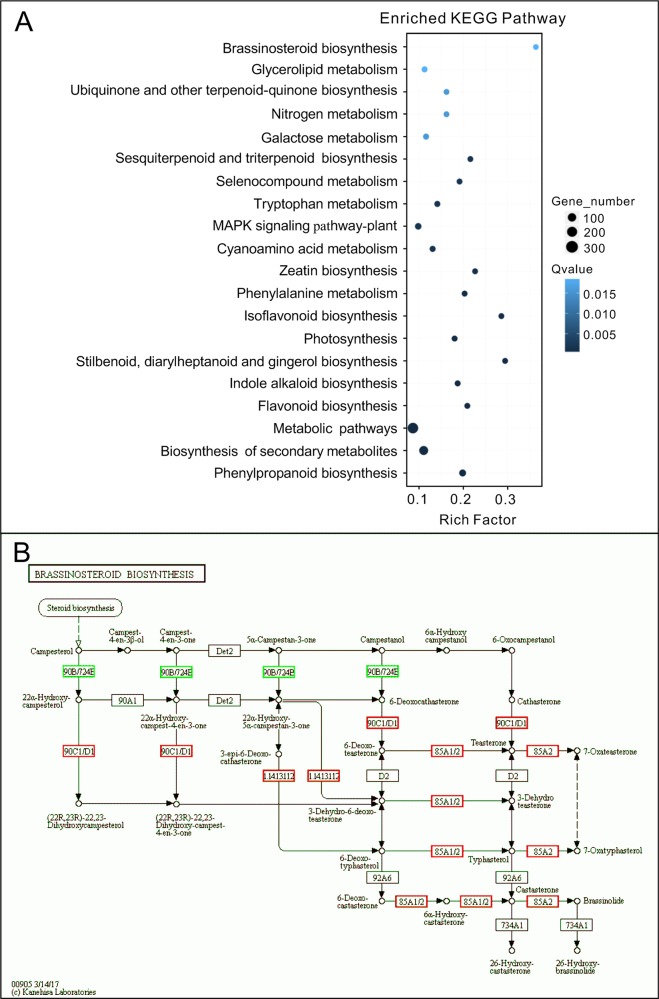
KEGG analysis of DEGs affected by melatonin. **(A)** The top 20 of the most significantly enriched KEGG pathways (*q*-value < 0.05). **(B)** Expression changes of genes in the brassinosteroid biosynthesis pathway. Red boxes, upregulated genes; green boxes, downregulated genes. KEGG, Kyoto Encyclopedia of Genes and Genomes; DEGs, differentially expressed genes.

To investigate the mechanism of hypocotyl elongation inhibition caused by melatonin, we performed a whole-genome expression profiling analysis between melatonin and DMSO treatment (DMSO as the solvent) by RNA sequencing. RNA-Seq results showed that there were 1,615 DEGs (7.47% of all unigenes), consisting of 776 upregulated DEGs and 839 downregulated DEGs (**Figure 1D**, **Supplementary Table S1**). The GO enrichment analysis showed that the DEGs were categorized into 68 significantly (*q*-value < 0.01) enriched GO terms including 17 terms of cellular component in which typical terms in respect to cell elongation, such as “cell wall” and “plant-type cell wall,” were included (**Supplementary Table S5**). In the top 30 of the most enriched GO terms, there were 11 terms involved in cellular component (**Figure 1E**). As far as the number of genes are concerned, many DEGs were enriched in cellular component ontology following the terms of “membrane part (504),” “intrinsic components of membrane (497),” and “integral component of membrane (466),” etc. (**Figure 1E**). Furthermore, the KEGG pathway analysis showed that the majority of DEGs were mainly distributed on 26 significantly enriched KEGG pathways (*q*-value < 0.05), such as “Phenylpropanoid biosynthesis,” “Biosynthesis of secondary metabolites,” and “Metabolic pathways” (**Figure 2A**, **Supplementary Table S6**). In addition, we found that “brassinosteroid biosynthesis” pathway displayed in the top 20 of most significantly enriched KEGG pathways (**Figure 2A**), and BR biosynthesis-related genes, such as three cytochrome P450 superfamily genes (*CYP724A1*, *CYP90D1*, and *BR6OX2*) and one abietane diterpene oxidase gene *ADTO1* (Fujioka and Yokota, 2003), showed differential expression (**Figure 2B**, **Table 1**), suggesting that melatonin affects the BR metabolism.

**Table 1:** Differentially expressed genes involved in brassinosteroid biosynthesis and cell elongation.

Gene ID	Symbol	Log2FC	*q*-value
**Brassinosteroid biosynthesis**			
AT3G13730	Cytochrome P450, family 90, subfamily D, polypeptide 1 (CYP90D1)	1.02	0.000000
AT3G30180	Brassinosteroid-6-oxidase 2 (BR6OX2)	1.66	0.000000
AT5G14400	Cytochrome P450, family 724, subfamily A, polypeptide 1 (CYP724A1)	−4.58	0.000431
AT1G73340	Abietane diterpene oxidase 1 (ADTO1)	−1.01	0.000023
**Cell elongation/expansion**			
AT5G39860	Paclobutrazol resistance1 (PRE1)	−1.30	0.000000
AT3G15540	Indole-3-acetic acid inducible 19 (IAA19)	−1.61	0.000000
AT1G52830	Indole-3-acetic acid inducible 6 (IAA6)	−1.55	0.000001
AT1G15050	Indole-3-acetic acid inducible 34 (IAA34)	−1.95	0.000000
AT2G01200	Indole-3-acetic acid inducible 32 (IAA32)	−1.04	0.000000
AT4G34790	Small auxin upregulated RNA 3 (SAUR3)	−1.42	0.000000
AT2G21200	Small auxin upregulated RNA 7 (SAUR7)	−1.13	0.000005
AT4G36110	Small auxin upregulated RNA 9 (SAUR9)	−1.46	0.000000
AT4G38825	Small auxin upregulated RNA 13 (SAUR13)	−1.81	0.000000
AT5G18010	Small auxin upregulated RNA 19 (SAUR19)	−1.62	0.000000
AT5G18020	Small auxin upregulated RNA 20 (SAUR20)	−1.23	0.000000
AT5G18030	Small auxin upregulated RNA 21 (SAUR21)	−1.07	0.000000
AT5G18050	Small auxin upregulated RNA 22 (SAUR22)	−1.84	0.000000
AT5G18060	Small auxin upregulated RNA 23 (SAUR23)	−1.30	0.000000
AT5G18080	Small auxin upregulated RNA 24 (SAUR24)	−1.25	0.000000
AT4G13790	Small auxin upregulated RNA 25 (SAUR25)	−2.36	0.000000
AT3G03820	Small auxin upregulated RNA 29 (SAUR29)	−1.40	0.000000
AT4G22620	Small auxin upregulated RNA 34 (SAUR34)	−1.34	0.000321
AT2G28085	Small auxin upregulated RNA 42 (SAUR42)	−2.11	0.000254
AT1G79130	Small auxin upregulated RNA 40 (SAUR40)	−1.65	0.000061
AT1G29430	Small auxin upregulated RNA 62 (SAUR62)	−1.38	0.000000
AT1G29440	Small auxin upregulated RNA 63 (SAUR63)	−1.29	0.000000
AT1G29460	Small auxin upregulated RNA 65 (SAUR65)	−1.34	0.000000
AT5G20820	Small auxin upregulated RNA 76 (SAUR76)	−1.06	0.000000
AT4G13390	Extensin 12 (EXT12)	1.01	0.000000
AT1G21310	Extensin (EXT3)	−1.48	0.000000
AT2G43150	Proline-rich extensin-like family protein	−1.74	0.000000
AT3G28550	Proline-rich extensin-like family protein	−1.34	0.000000
AT3G24480	Proline-rich extensin-like family protein	−1.24	0.000000
AT1G65310	Xyloglucan endotransglucosylase/hydrolase 17 (XTH17)	−1.17	0.000000
AT3G23730	Xyloglucan endotransglucosylase/hydrolase 16 (XTH16)	−1.01	0.000000
AT4G25810	Xyloglucan endotransglycosylase 6 (XTR6)	1.04	0.000000
AT4G28850	Xyloglucan endotransglucosylase/hydrolase 26 (XTH26)	1.19	0.000000
AT3G48580	Xyloglucan endotransglucosylase/hydrolase 11 (XTH11)	1.47	0.000000
AT1G70720	Plant invertase/pectin methylesterase inhibitor superfamily protein	−2.52	0.000000
AT2G45220	Pectin methylesterase 17 (ATPME17)	−2.05	0.000000
AT3G26610	Polygalacturonase involved in expansion 1 (PGX1)	−1.17	0.000000
AT5G55590	Quartet1 (QRT1)	2.38	0.000000
AT2G21610	Pectinesterase 11 (PE11)	1.67	0.000290
AT5G20860	Plant invertase/pectin methylesterase inhibitor superfamily	2.18	0.000000
AT3G12880	Plant invertase/pectin methylesterase inhibitor superfamily protein	2.55	0.000003
AT2G14900	Gibberellin-regulated family protein	−1.07	0.000000
AT5G14920	GA-stimulated in *Arabidopsis* 14 (GASA14)	−1.06	0.000000
AT1G22690	Gibberellin-regulated family protein	1.40	0.000000
AT4G23560	Glycosyl hydrolase 9B15 (GH9B15)	−2.82	0.000089
AT4G02290	Glycosyl hydrolase 9B13 (GH9B13)	−1.16	0.000000
AT2G32990	Glycosyl hydrolase 9B8 (GH9B8)	1.37	0.000000
AT1G24070	Cellulose synthase-like A10 (CSLA10)	−1.47	0.000003
AT4G24000	Cellulose synthase-like G2 (CSLG2)	1.73	0.000000
AT4G38080	Hydroxyproline-rich glycoprotein family protein	2.50	0.000000
AT2G36970	UDP-glycosyltransferase superfamily protein	1.61	0.000000

Differentially expressed genes were defined by two-fold change difference between melatonin and dimethyl sulfoxide treatment with q-value ≤ 0.001 (Storey and Tibshirani, 2003).

A further analysis showed that there were 52 DEGs in respect to cell elongation/expansion. Among these DEGs, most of them (38 out of 51) were downregulated and only 13 DEGs were upregulated (**Table 1**). In addition, *SAUR* genes involved in auxin response and functions on the regulation of cell elongation and cell expansion (Knauss et al., 2003; Jain and Khurana, 2009; Ren and Gray, 2015) accounted for 37% (19 out of 51) and displayed consistent downregulated expression (**Table 1**). In these *SAUR*s, most of them, such as *SAUR15*, *SAUR16*, and *SAUR19* to *SAUR24*, have also been proven to respond to BR signaling in the regulation of hypocotyl growth (Favero et al., 2017). Furthermore, the BR-induced genes as well the marker genes like the *PRE1* and *indole-3-acetic acid inducible 19* (*IAA1*9) also displayed decreasing transcript levels (**Table 1**). These results suggest that a high concentration of melatonin represses hypocotyl elongation in relation to reprograming cell elongation/expansion genes expression likely through regulating BR metabolism.

### Melatonin Regulates BR-Related Genes Expression

**Figure 3:**
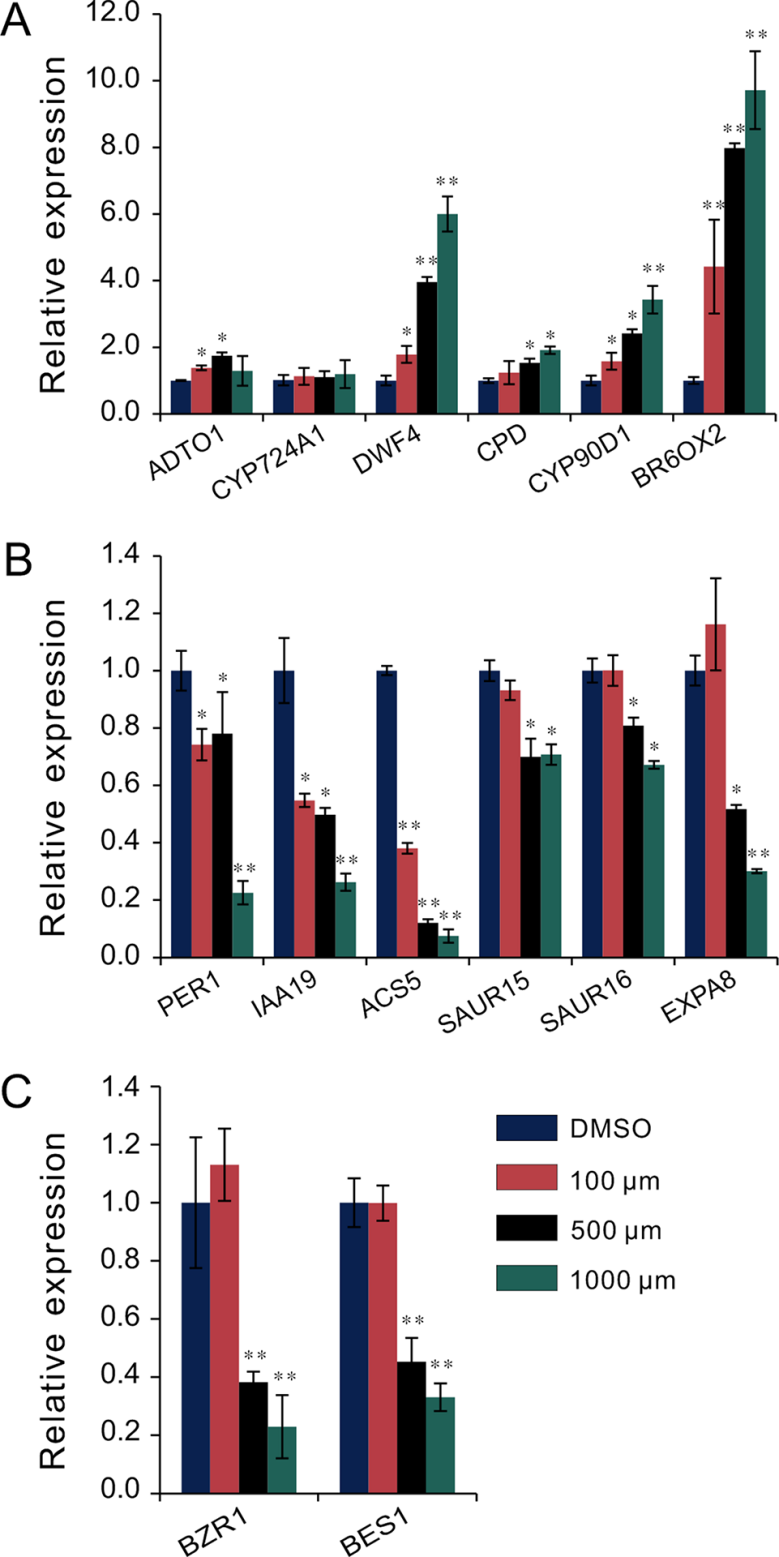
qRT-PCR analysis of BR-related gene expression under melatonin treatment. Seedlings were grown in light for 4 days and then transferred to 1/2MS plates with various concentrations of melatonin in a dark condition for another 3 days. Expression levels were normalized to an internal control *ACTIN2*. **(A)** qRT-PCR analysis of BR-biosynthetic genes. **(B)** qRT-PCR analysis of BR-induced genes involved in cell elongation. **(C)** qRT-PCR analysis of the two key transcription factors, *BZR1* and *BES1*. Data are represented as means ± SD (*n* = 3), *p < 0.05, **p < 0.01 (Student’s *t* test). BR, brassinosteroid; MS, Murashige and Skoog; qRT-PCR, real-time polymerase chain reaction.

To investigate the regulatory relationship between melatonin and BR signaling, we screened the BR biosynthesis-related genes and representative BR-induced genes involved in cell elongation in the total detected genes of RNA-Seq data. Except of *CYP724A1*, *CYP90D1*, *BR6OX2*, and *ADTO1*, the key rate-limiting enzyme gene *CPD* in BR biosynthesis also displayed differential upregulated expression at 1.5-fold change threshold (**Supplementary Table S1**). Conversely, in addition to *PER1* and *IAA19*, BRs-induced as well as marker genes regulating cell elongation, like the *SAUR15*, *SAUR16*, and *EXPA8*, exhibited consistent downregulated expression with a 1.5-fold change threshold. Furthermore, *ACS5*, usually taken as a marker gene in response to BRs, exhibited reduced transcript level as well (**Table 1**, **Supplementary Table S1**). We next performed qRT-PCR to validate the expression levels of these BR-biosynthetic and BR-induced genes in the seedlings after being treated with various concentrations of melatonin. Except of the two genes *CYP724A1* and *ADTO1*, which showed smooth expression levels, a consistent trend of expression was obtained with these detected genes (**Figure 3A**). BR-biosynthetic genes (*DWF4*, *CPD*, *CYP90D1*, and *BR6OX2*) showed dramatic enhancement with increasing concentrations of melatonin, especially those of *DWF4* and *BR6OX2* (**Figure 3A**)*.* On the contrary, the BR-induced genes (*PER1*, *IAA19*, *ACS5*, *SAUR15*, *SAUR16*, and *EXPA8*) displayed an identical decline in transcript level caused by increasing dose of melatonin (**Figure 3B**). Additionally, we also detected the transcript levels of BZR1 and BES1, two key transcription factors in BR signaling, which promote cell elongation through transcriptional activation of BR-induced genes, such as *PER1*, *IAA19*, *SAUR*s, and *EXPA8*, whereas promote feedback suppression in the expression of BR-biosynthetic genes (like the *DWF4*, *CPD*, and *BR6OX2*) (Sun et al., 2010; Yu et al., 2011; Bai et al., 2012; Wang et al., 2012; Nolan et al., 2017). Consistent with BR-induced genes, *BZR1* and *BES1* also showed downregulated trend in transcript levels with increasing dose of melatonin (**Figure 3C**). These results are consistent with the gene expression of the inhibition of BR signaling, implying that a high dose of melatonin represses BR signaling pathway in the regulation of hypocotyl elongation.

### Melatonin Inhibits Hypocotyl Elongation by Repressing BR Signaling

**Figure 4:**
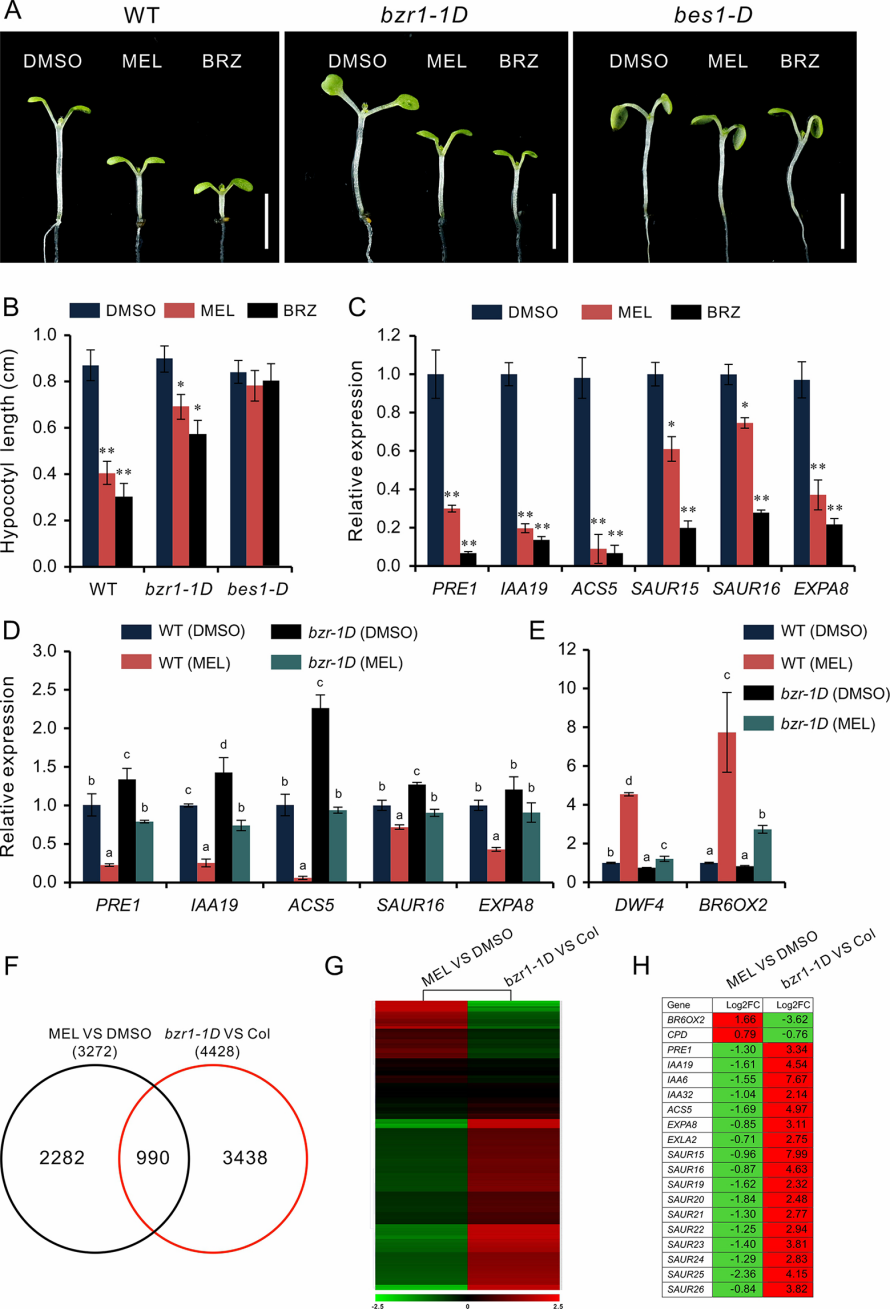
High-dose melatonin represses BR signaling. **(A, B)** Four-day-old seedlings of WT, *bzr1-1D*, and *bes1-D* were transferred on medium containing 1 mM of melatonin (MEL), 1 µM brassinazole (BRZ), or 0.1% DMSO (solvent control) for 3 days in darkness. Scale bar represents 0.5 cm. *p < 0.05, **p < 0.01 (Student’s *t* test). Error bars indicate the SEM (*n* ≥ 40). **(C)** qRT-PCR analyses of expression of BR-related genes in WT seedlings under treatment of MEL, BRZ, and DMSO (control). Data are represented as means ± SD (*n* = 3), *p < 0.05, **p < 0.01 (Student’s *t* test). **(D, E)** Expression level analysis of BR-related genes in both WT and *bzr1-1D* after being treated with melatonin. Significant differences (*p* < 0.05) are denoted by different lowercase letters. **(F)** Venn diagram displaying the overlapping genes regulated by melatonin and BZR1. **(G, H)** Hierarchical cluster analysis of 990 overlapping genes and the representative genes in BR signaling are listed. Red and green colors in the heatmap represent induced and repressed genes, respectively. BR, brassinosteroid; DMSO, dimethyl sulfoxide; qRT-PCR, real-time polymerase chain reaction; SEM, standard error of the mean; WT, wild type.

Based on the above observations, we speculated that melatonin inhibits hypocotyl likely by direct repression of BR signaling. This idea was supported by the melatonin sensitivity experiment performed on *bzr1-1D* and *bes1-D*, which are BR constitutive active form mutants of *BZR1* and *BES1*, respectively. Similar with the BR biosynthetic inhibitor BRZ, melatonin markedly (*p* < 0.01) inhibited hypocotyl elongation of WT but showed slight (*p* < 0.05) suppression on *bzr1-1D* and had no impact on hypocotyl elongation of *bes1-D* (**Figures 4A, B**). Furthermore, an expression analysis also showed the same tendency in downregulation of BR-induced genes (*PRE1*, *IAA19*, *ACS5*, *EXP8*, *SAUR15*, and *SUAR16*) between melatonin and BRZ (**Figure 4C**), suggesting that high concentrations of melatonin are equivalent to the BRZ in the inhibition of hypocotyl elongation. We also analyzed the expression of these genes in *bzr1-1D* seedlings after being treated with melatonin and found that these genes were poorly affected by melatonin as compared to those of WT control (**Figure 4D**). In addition, the upregulation of *DWF4* and *BR6OX2* caused by melatonin was partly suppressed in *bzr1-1D* (**Figure 4E**).

We then carried out a comparative transcription analysis between the data of MEL versus DMSO and that of *bzr1-1D* versus Col, which was obtained from public RNA-Seq data online (**Supplementary Table S2**) (Tian et al., 2018). We found that of 3,272 DEGs regulated by melatonin, 990 genes (30%) were shared by the DEGs (4,428) in *bzr1-D* versus Col group (**Figure 4F**). Heatmap showed an opposite pattern of expression among these overlapping genes, in which 705 genes (71%) were affected in the opposite way (**Figure 4G**). In particular, the marker genes of BR signaling, such as BR-biosynthetic genes (*BR6OX2* and *CPD*) and BR-induced gens (*PER1*, *IAA19*, *EXPA8*, *SAUR*s, etc) involved in cell elongation, also displayed an opposite expression trend (**Figure 4H**), suggesting that the inhibition of hypocotyl elongation caused by melatonin involves the repression of BR signaling.

### BL Antagonizes Melatonin in the Regulation of Hypocotyl Elongation

**Figure 5:**
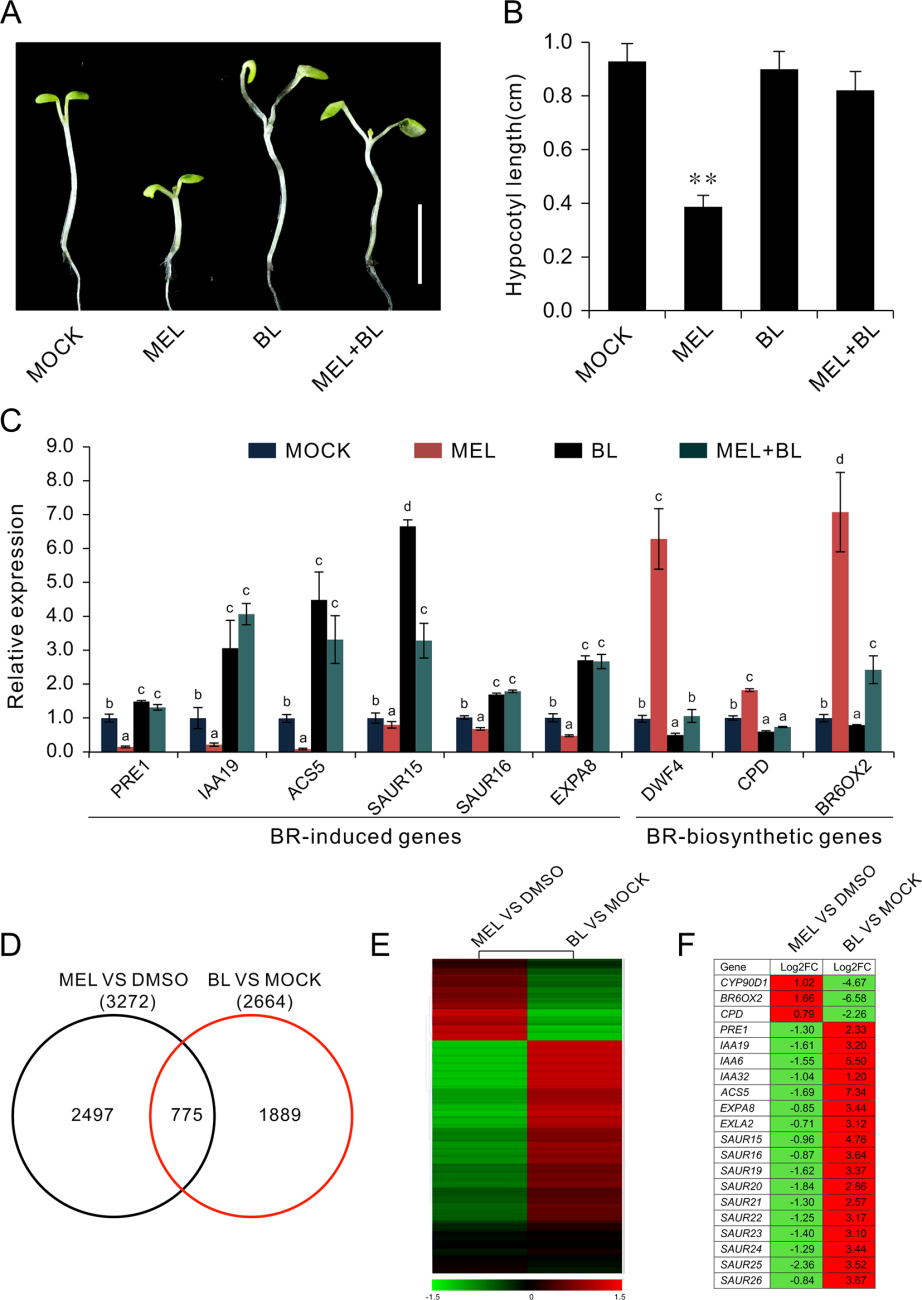
BL antagonizes melatonin in the regulation of hypocotyl elongation. **(A)** Additional BL restores hypocotyl elongation against a high concentration of melatonin. Four-day-old seedlings grown on 1/2MS under light were transferred on medium containing 1 mM of melatonin with indicated concentration of BL for another 3 days in darkness. Scale bar represents 0.5 cm. **(B)** The corresponding hypocotyl length.*p < 0.05, **p < 0.01 (Student’s *t* test). Error bars indicate the SEM (*n* ≥ 40). **(C)** qRT-PCR analyses showing the expression of BR-related genes. Significant differences (*p* < 0.05) are denoted by different lowercase letters. **(D)** Venn diagram displaying the overlapping genes induced by melatonin and BL. **(E, F)** Hierarchical cluster analysis of 775 overlapping genes and the representative genes in BR signaling are listed. Red and green colors in the heatmap represent induced and repressed genes, respectively. BL, brassinolide; MS, Murashige and Skoog; qRT-PCR, real-time polymerase chain reaction; SEM, standard error of the mean.

Exogenous BL is usually used to rescue dwarf hypocotyl of BR biosynthesis synthesis mutants (Wang et al., 2002; De Rybel et al., 2009). To test the relationship between the melatonin and BR pathways, we transferred the seedlings to melatonin-enriched medium supplemented with various concentrations of BL. The results showed that hypocotyl inhibition caused by high dose of melatonin was gradually restored by additional BL, but not IAA or GA_3_ (**Figures 5A, B**, and **Supplementary Figure S2**). Consistent with the retrieval of *bzr1-1D* and *bes1-D* on hypocotyl repression caused by melatonin (**Figure 4A**), the inhibitory effects of 1 mM of melatonin on hypocotyl were almost eliminated by 1 μM BL (**Figures 5A, B**). Furthermore, an expression analysis of BR-related genes showed that BL antagonized the high dose of melatonin in the regulation of the expression of BR-related genes. Concretely, BL increased the expression of BR-induced genes (*PRE1*, *IAA19*, *ACS5*, *SAUR15*, and *SUAR16*) but reduced the expression of BR-biosynthetic genes (*DAWF4*, *CPD*, and *BR6OX2*), even in the presence of 1 mM of melatonin (**Figure 5C**), implying that melatonin affects BR biosynthesis.

To further explore the regulatory relationship between melatonin and BR signaling, we compared the 3,272 DEGs (1.5-fold change) affected by melatonin (MEL versus DMSO) with the 2,664 DEGs induced by BL (BL versus mock) from public RNA-Seq data (**Supplemental Table S3**) (Oh et al., 2014) and found that of 3,272 melatonin regulated DEGs, and 775 genes (24%) were also affected by BL (**Figure 5D**, **Supplementary Table S3**). Among these co-regulated genes, 544 genes (70%) in MEL versus DMSO group were downregulated, whereas in BL versus mock group, most of the genes (492 out of 775) were upregulated (**Supplementary Table S3**). Heatmap also showed an opposite regulated mode where 597 (77%) genes were affected in an opposite way between melatonin and BL (**Figure 5E**, **Supplementary Table S3**). In addition, some representative genes such as BR-biosynthetic genes (*CYP90D1*, *BR6OX2*, and *CPD*) and BR-induced genes (*PER1*, *IAA19*, *ACS5*, *EXPA8*, *SAUR15*, *SAUR16*, etc.) of the co-regulated genes exhibited reverse expression mode (**Figure 5F**). In addition, similar results were observed when genes affected by melatonin (MEL versus DMSO) were compared with the genes induced by BL (BL versus mock) and the genes regulated in *bzr1-1D* (*bzr1-1D* versus Col) (**Supplementary Figure S3**).

### Melatonin Inhibits BR Biosynthesis

**Figure 6:**
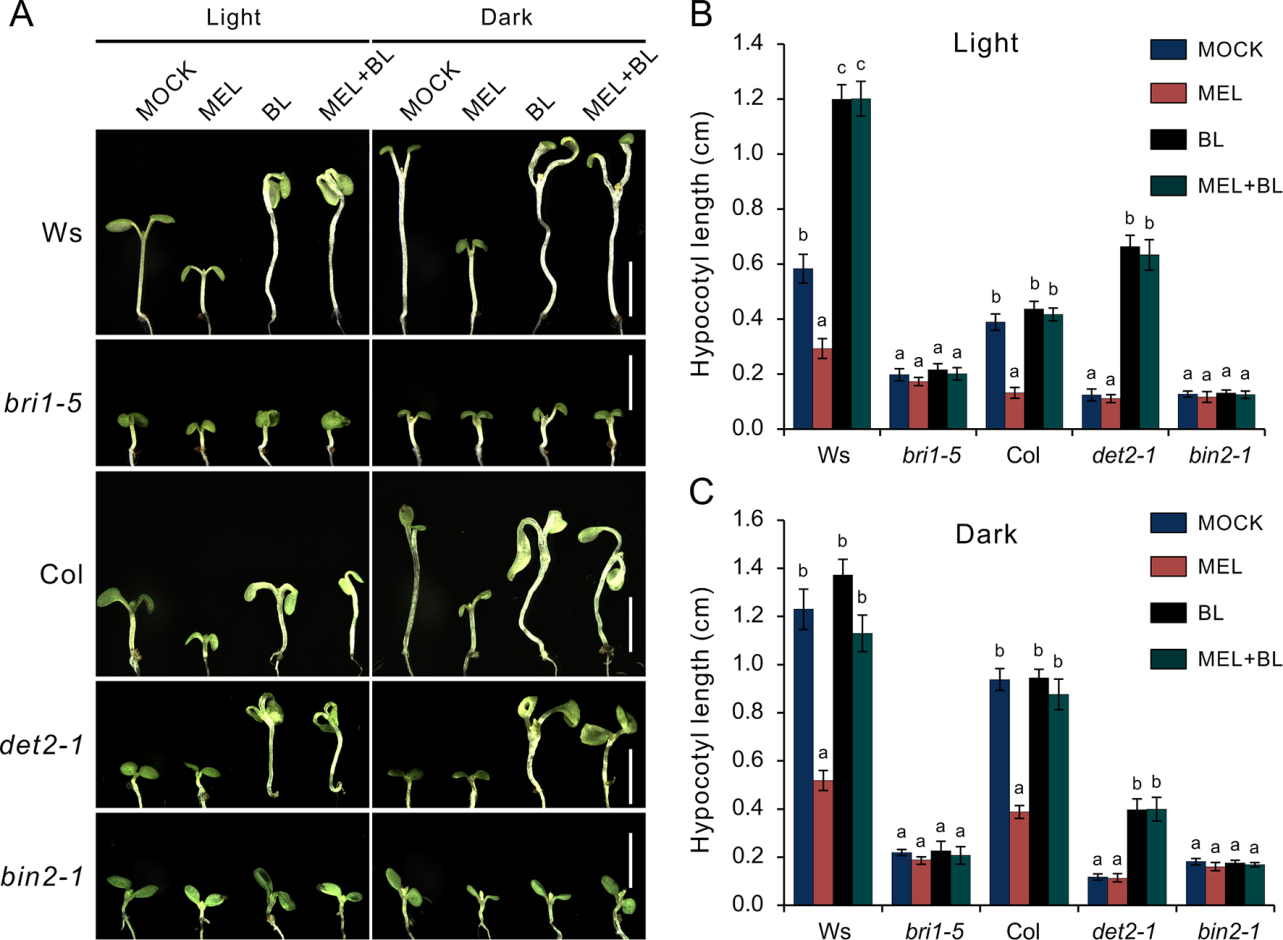
Melatonin involves in repressing BR biosynthesis. **(A)** Phenotype of BR-related mutants like the *det2-1*, *bri1-5*, and *bin2-1* response to 1 mM of melatonin, 1 µM of BL or their combination, and mock solvent as the control. Scale bar represents 0.5 cm. **(B, C)** Hypocotyl length of the corresponding seedlings. Error bars indicate the SEM (*n* ≥ 40). Significant differences (*p* < 0.05) are denoted by different lowercase letters. BL, brassinolide; BR, brassinosteroid; SEM, standard error of the mean.

**Figure 7:**
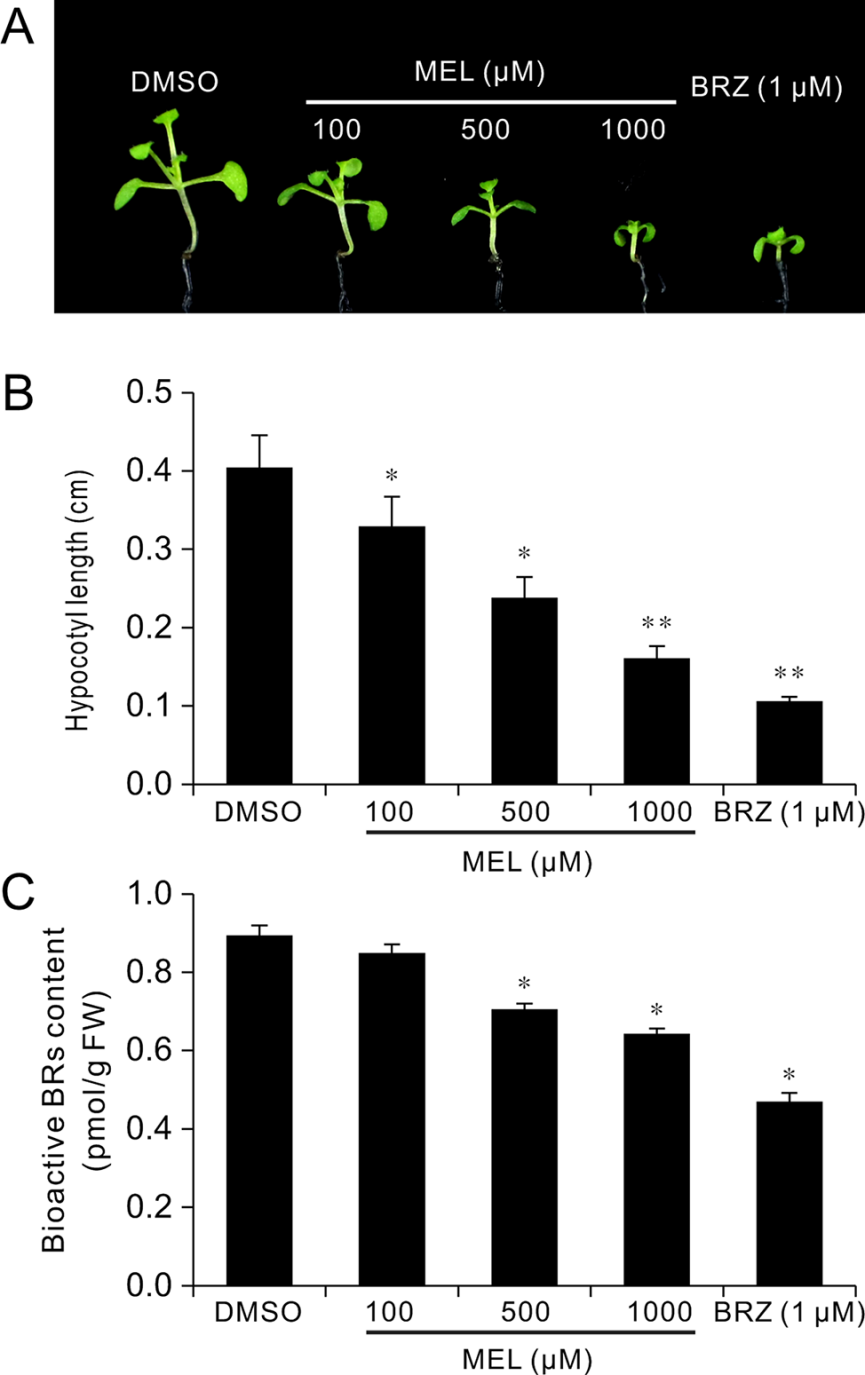
Melatonin reduces endogenous BR levels. Seedlings 12 DAG from 1/2MS amended with melatonin, BRZ, or DMSO (control) were collected for the determination of BR levels by ELISA method. **(A)** Phenotype of seedlings from melatonin, BRZ, or DMSO treatments. **(B)** Hypocotyl length of the corresponding seedlings. *p < 0.05, **p < 0.01 (Student’s *t* test). Error bars indicate the SEM (*n* ≥ 40). **(C)** Endogenous bioactive BR content. Data are represented as means ± SD (*n* = 3), *p < 0.05 (Student’s *t* test). BR, brassinosteroid; BRZ, brassinazole; DAG, days after germination; DMSO, dimethyl sulfoxide; ELISA, enzyme-linked immunosorbent assay; MS, Murashige and Skoog; SEM, standard error of the mean.

To explore how melatonin acts on BR signaling, the effects of melatonin on BR-deﬁcient mutants were also investigated, such as BR synthesis mutant *det2-1*, the BR receptor mutant *bir1-5*, and BR-insensitive 2 mutant *bin 2-1.* The results showed that exogenous melatonin had little effect on hypocotyl elongation of these BR-deﬁcient mutants compared with the control whether they were under normal light or dark conditions (**Figure 6A**)*.* It has been report that additional BL can repress a BR-deﬁcient phenotype of BR synthesis mutants (such as *det2*, *cpd*, and *dwarf4*), but not to BR receptor mutants or other mutants involved in BR signal transduction (Wang et al., 2002; De Rybel et al., 2009). Consistent with previous reports, exogenous BL dramatically promoted hypocotyl elongation of *det2-1* but not *bri1-5* and *bin2-1*, even in presence of a high dose of melatonin (**Figures 6A–C**). Furthermore, we measured the bioactive BR contents by ELISA method. Consistent with the hypocotyl inhibition, a high concentration of melatonin reduced endogenous BR levels to a similar extent with biosynthetic inhibitor BRZ (**Figures 7A–C**). These results further confirmed that a high dose of melatonin repressed BR biosynthesis in the inhibition of hypocotyl elongation.

## Discussion

Melatonin functions as a pleiotropic regulator in the regulation of plant growth, development, and multiple stress responses (Hardeland et al., 2011; Arnao and Hernández-Ruiz, 2014; Arnao and Hernández-Ruiz, 2015; Erland et al., 2015; Nawaz et al., 2016; Kanwar et al., 2018). In terms of dosage melatonin, the working concentration of melatonin is usually considerably higher than that of other plant hormones in the study of plant growth and development. For example, in the research of how melatonin regulates *Arabidopsis* root system architecture, the maximum concentration of melatonin (600 μM) was used (Pelagio-Flores et al., 2013); in another study, melatonin regulates the floral transition in *Arabidopsis*, and up to 1 mM of melatonin was applied (Shi et al., 2016). In addition, a high concentration of melatonin (1 mM) was used to investigate its inhibitory effects on leaf development in *Arabidopsis* (Wang et al., 2017). Growth retardations caused by a relatively high concentration of exogenous melatonin have been reported in many studies. It has been reported that melatonin at a high concentration shows an inhibitory effect on hypocotyl elongation of etiolated seedlings in lupin (Hernandez-Ruiz et al., 2004). A recent study showed us that high-concentration melatonin restrained root growth and reduced biomass, as well as retarded plant leaf growth by reducing both cell size and cell proliferation (Wang et al., 2017; Lin et al., 2018). There were many investigations showing that high-dose melatonin accompanied inhibitory effects; however, the molecular mechanism remains largely unknown. In present study, we found that a high dose of melatonin inhibited hypocotyl and cell elongation (**Supplementary Figure S1**, **Figures 1A–C**) and mechanistically revealed that hypocotyl inhibition of melatonin is involved in the repression of BR biosynthesis in *Arabidopsis*.

Our data revealed that there were 1,615 DEGs affected by melatonin, in which many genes involved in cell elongation/expansion were downregulated (**Figure 1D**, **Table 1**, **Supplementary Table S1**). This also explains why a high dose of melatonin inhibits hypocotyl elongation. There are several literatures described that melatonin participates in regulating gene expression in respect with plant hormones including auxin, cytokinin, GAs, abscisic acid, ethylene, jasmonic acid, and salicylic acid (Arnao and Hernández-Ruiz, 2018; Arnao and Hernández-Ruiz, 2019). Melatonin showed effects on inducing expression of phytohormone related-genes such as biosynthesis, catabolism, receptors, and transcription factors (Arnao and Hernández-Ruiz, 2018; Arnao and Hernández-Ruiz, 2019). Our KEGG pathway analysis also found that “brassinosteroid biosynthesis” pathway listed in the top 20 of most significantly enriched KEGG pathways and BR biosynthesis-related genes showed differential expression (**Figures 2A, B**, **Table 1**), suggesting that melatonin affects the BR metabolism. It is well known that BRs play a predominant role in the regulation of cell elongation; thus, we speculated that hypocotyl inhibition of melatonin is likely to be involved in the regulation of BR signaling. Our results further showed that melatonin modulated BR-related genes expression, such as downregulating BR-induced genes involved in cell elongation (*PRE1*, *IAA19*, and *SAUR*s) but upregulating BR-biosynthetic genes (*DAWF4*, *CPD*, and *BR6OX2*) (**Figures 3A, B**, **Table 1**). In BR signaling, BRs activated and stabilized the two key transcription factors, BZR1 and BES1, to promote cell elongation through transcriptional activation of their target genes involved in cell elongation, including *PER1*, *IAA19*, *SAUR*s, and *EXPA8* (Sun et al., 2010; Yu et al., 2011; Bai et al., 2012; Wang et al., 2012, Nolan et al., 2017). Therefore, it is easy to understand that constitutive active forms of mutants *bzr1-1D* and *bes1-D* showed insensitivity to BRZ as well as a high dose of melatonin (**Figures 4A–D**). Through a comparative expression analysis, we further confirmed that melatonin represses BR signaling in regulating hypocotyl elongation (**Figures 4F–H** and **5D–F** and **Supplementary Figure S3**).

We also observed that an inhibitory effect on hypocotyl of melatonin was eliminated by exogenous BL (**Figures 5A–C**, **Supplementary Figure S2**), implying that melatonin functions in BR biosynthesis, because an exogenous application of BL could recover hypocotyl elongation of a BR-deﬁcient phenotype of BR synthesis mutants (*det2*, *cpd*, *dwarf4*, etc.), but not to BR receptor mutants (*bri1-5*) or other mutants (*bin2-1*) involved in BR signal transduction (Wang et al., 2002; De Rybel et al., 2009). In the present study, we also observed that even in the presence of high concentrations of melatonin, BL reversed hypocotyl inhibition of WT and *det2-1* mutant seedlings, but not *bri1-5* or *bin2-1* mutants (**Figures 6A–C**). These observations further confirmed that melatonin suppresses BR biosynthesis in the regulation of hypocotyl elongation. In addition, the results of quantification of bioactive BR contents under treatment with melatonin also supported our conclusions (**Figures 7A–C**). On the other hand, the recent investigations reported that rice seedlings with melatonin deficiency by knockdown of the enzyme gene required for melatonin biosynthesis showed a semi-dwarf phenotype and reduction of *DWARF4* transcripts and reduction of endogenous BR levels (Hwang and Back, 2018; Lee and Back, 2018), suggesting that the lack of melatonin synthesis is likely related to BR suppression. In the present study, contradicting results were obtained likely due to the difference in species, because of no BR-deficient phenotype; for example, a de-etiolated phenotype in the dark condition and a short hypocotyl in the light condition were observed in melatonin biosynthesis-deficient mutants such as *snat1* (Lee and Back, 2018).

BRs and GAs are two primary hormones regulating plant cell elongation/expansion; additionally, auxin also plays an important role in regulating cell elongation (Depuydt and Hardtke, 2011; Bai et al., 2012). It has been reported that melatonin upregulated GA biosynthesis genes such as *GA20ox* and *GA3ox* in cucumber seedlings in response to saline stress and resulted in a high level of active GAs such as GA3 and GA4 in the salt-inhibited germination process (Zhang et al., 2013; Zhang et al., 2014). Recently, melatonin was reported to negatively regulate auxin biosynthesis and the auxin response in *Arabidopsis* (Wang et al., 2016). Together, the above findings imply that melatonin inhibited hypocotyl elongation likely involved in the regulation of GA or auxin signaling. The expression profile analysis also showed a certain amount of auxin response genes, such as *SAUR*s and *IAAs*, in which some are co-regulated by BRs and auxin, involved in the regulation of cell elongation (**Table 1**). However, our results showed that just BL, but not IAA or GA_3_, could save the hypocotyl elongation (**Supplementary Figure S2**), suggesting that BRs, not auxin and GAs, play a predominant role in mediating the inhibition of hypocotyl elongation caused by a high concentration of melatonin.

Based on our results, we propose that melatonin decreases endogenous BR content through repressing BR biosynthesis, which leads to inactivation of BR signaling and reduction of transcriptional activity of BZR1/BES1 and then downregulation of the expression of BR-induced genes (*PRE1*, *IAA19*, *EXPA8* and *SAUR*s) involved in hypocotyl cell elongation and upregulation of the expression of BR-biosynthetic genes (*DAWF4*, *CPD*, *CYP90D1*, and *BR6OX2*) by negative feedback regulation. In summary, our findings reveal that melatonin inhibits hypocotyl elongation through repression of the BR biosynthesis in *Arabidopsis*; however, the mechanism of action needs further study to investigate how melatonin acts in the biosynthesis of BRs.

## Data Availability

All datasets for this study are included in the manuscript and the **Supplementary Files**.

## Author Contributions

FX and FZ designed the experiments; FX, ZW, and LF performed the experiments; KD, YS, LW, and GQ analyzed the data; FX and MR wrote the manuscript; and RR, ZY, and FL revised the manuscript and provided valuable advice.

## Funding

This work was supported by the following grants: the National Natural Science Foundation of China (Nos. 31801913 and 31801271), the Major Program of Joint Funds (Henan, Grant No. U1804231), the China Postdoctoral Science Foundation (No. 2017M622958), and the Fundamental and Frontier Research Project of Chongqing (No. cstc2016jcyjA0822).

## Conflict of Interest Statement

The authors declare that the research was conducted in the absence of any commercial or financial relationships that could be construed as a potential conflict of interest.

## Supplementary Material

Supplementary Figure S1 Exogenous melatonin inhibits seedling growth in Arabidopsis.

Supplementary Figure S2 Additional BL rescued hypocotyl elongation under melatonin treatment

Supplementary Figure S3 Comparative expression spectrum analysis

Supplementary Table S1 Differentially expressed genes between melatonin treatment and DMSO control (MEL VS DMSO).

Supplementary Table S2 Comparative expression spectrum analysis between MEL VS DMSO and bzr1-1D VS Col.

Supplementary Table S3 Comparative expression spectrum analysis between MEL VS DMSO and BL VS Mock.

Supplementary Table S4 Primers used for qRT-PCR.

Supplementary Table S5 Enrichment of GO terms

Supplementary Table S6 Enrichment of KEGG pathways
