# Supplementary material for: Hypocotyl Elongation Inhibition of Melatonin Is Involved in Repressing Brassinosteroid Biosynthesis in Arabidopsis
Source: Front Plant Sci. 2019 Sep 26;10:1082. doi: 10.3389/fpls.2019.01082 (PMC6775476; doi:10.3389/fpls.2019.01082)
Supplement: Supplementary Figure S1 — Exogenous melatonin inhibits seedling growth in Arabidopsis. [file DataSheet_1.zip › Supplementary files/Supplementary Table S4 Primers used for qRT-PCR..docx]

Supplementary Table S4 Primers used for qRT-PCR.

| Gene | Accession no. | Forward (5’-3’) | Reverse (5’-3’) |
| --- | --- | --- | --- |
| *PRE1* | AT5G39860 | AATGATTGACCTCGTATCTAAG | TCTAATAACGGCGGCTTCAG |
| *IAA19* | AT3G15540 | CTCGGGCTTGAGATAACGG | TTCTCAGCGTCACCACCAG |
| *ACS5* | AT5G65800 | GCTATGGCTGAGTTTATGG | TTGAGCAGTGAATGGGTAC |
| *SAUR15* | AT4G38850 | TTGAGGAGTTTCTTGGGTGCT | ACAAAGGCTGGTTTAAGTATG |
| *SAUR16* | AT4G38860 | ATGCTACGACGAGGAAGGT | ATGACGAAAGCCGAACTCT |
| *EXPA8* | AT2G40610 | GCGTGCTATGAGATGAAGTG | ATGCTGAAGAGGAGGATTGC |
| *IAA19* | AT3G15540 | CTCGGGCTTGAGATAACGG | TTCTCAGCGTCACCACCAG |
| *ADTO1* | AT1G73340 | ACGCCGTCGCTGGTTCTCA | CTCAGTGTCTGGAGCATAACG |
| *CYP90D1* | AT3G13730 | CGGGAGTTTACATAGACGG | CTTGGAGGAGCACAGGTTG |
| *CYP724A1* | AT5G14400 | CATCAAGCCAGAAGACCCA | ATAACCCTCGCCGACAATC |
| *DWF4* | AT3G50660 | TCTTCAGTCACGAGCAACG | TCTCATGTCCGGCAAATAAC |
| *CPD* | AT5G05690 | CGTTTAGAGCGGTTCATTTAG | GTTTCTGCGTTCTTGTAGTTG |
| *BR6OX2* | AT3G30180 | CTGACGAACCGCTCACTCTC | GCAAAGCCTAACTCCACCTC |
| *BRZ1* | AT1G75080 | CAAGCCTTTACCTGGTGAGA | ACGCCATTGATTGTTTAGC |
| *BES1* | AT1G19350 | AAGACGGAACTACTTATCGC | CTGGTGGAGTGACAGGAGC |
| *ACTIN2* | AT3G18780 | GCACTTGCACCAAGCAGCAT | CCTTTCAGGTGGTGCAACGAC |
