# Supplementary figures and images for: Hypocotyl Elongation Inhibition of Melatonin Is Involved in Repressing Brassinosteroid Biosynthesis in Arabidopsis
Source: Front Plant Sci. 2019 Sep 26;10:1082. doi: 10.3389/fpls.2019.01082 (PMC6775476; doi:10.3389/fpls.2019.01082)

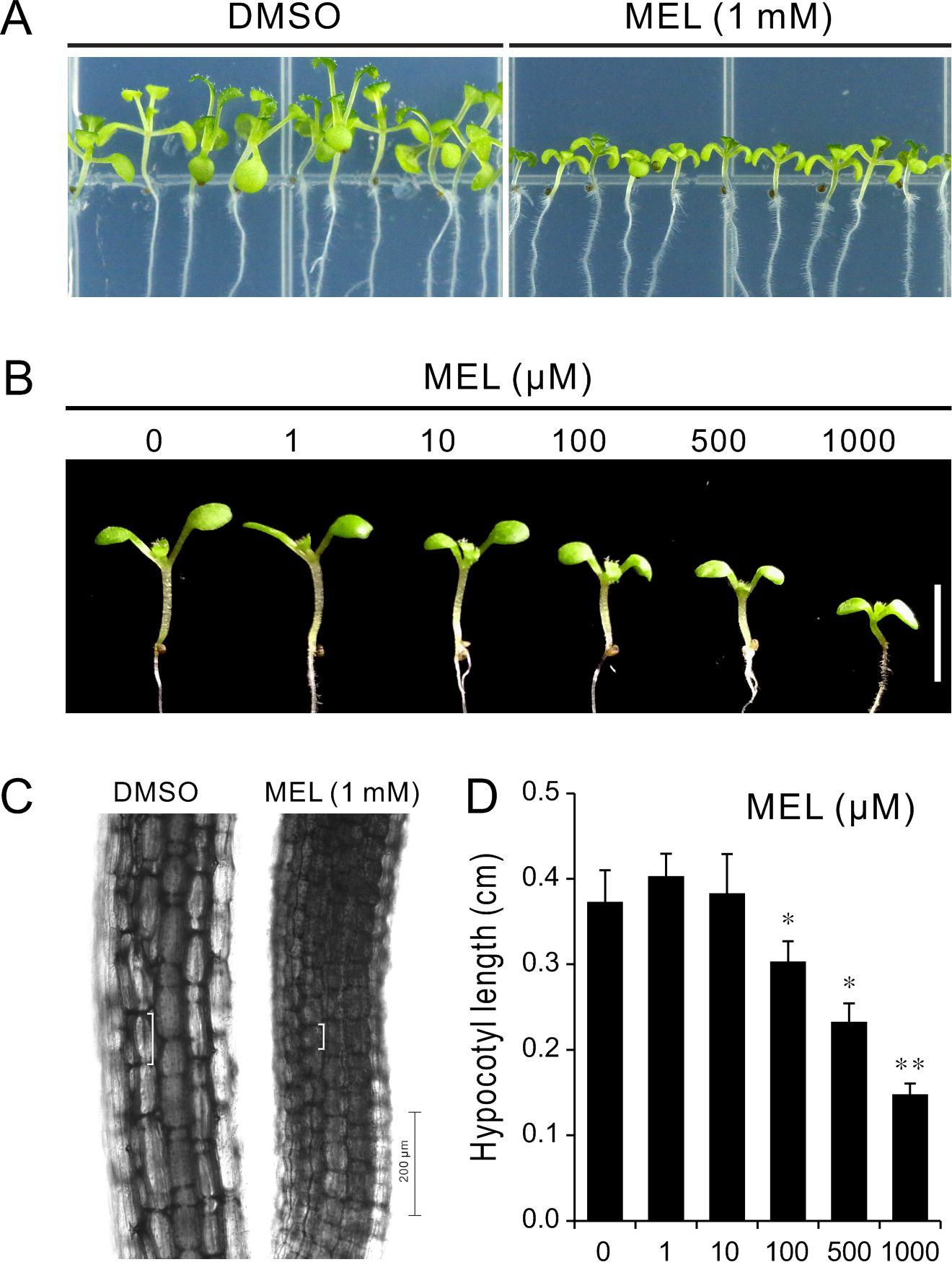

Supplement: Supplementary Figure S1 — Exogenous melatonin inhibits seedling growth in Arabidopsis. [file DataSheet_1.zip › Supplementary files/Supplementary Figure S1 Exogenous melatonin inhibits seedling growth in Arabidopsis..tif]

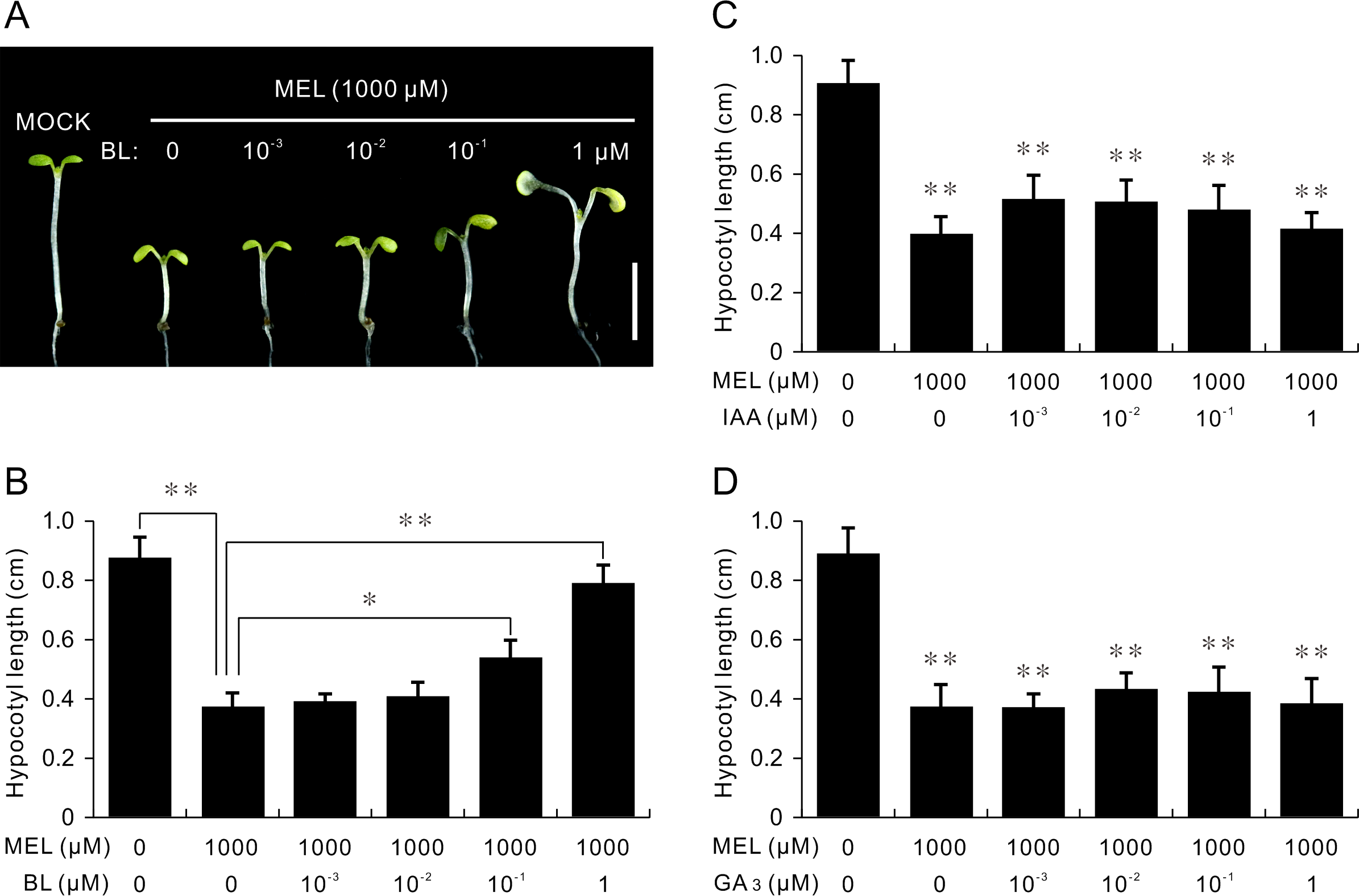

Supplement: Supplementary Figure S1 — Exogenous melatonin inhibits seedling growth in Arabidopsis. [file DataSheet_1.zip › Supplementary files/Supplementary Figure S2 Additional BL rescued hypocotyl elongation under melatonin treatment.tif]

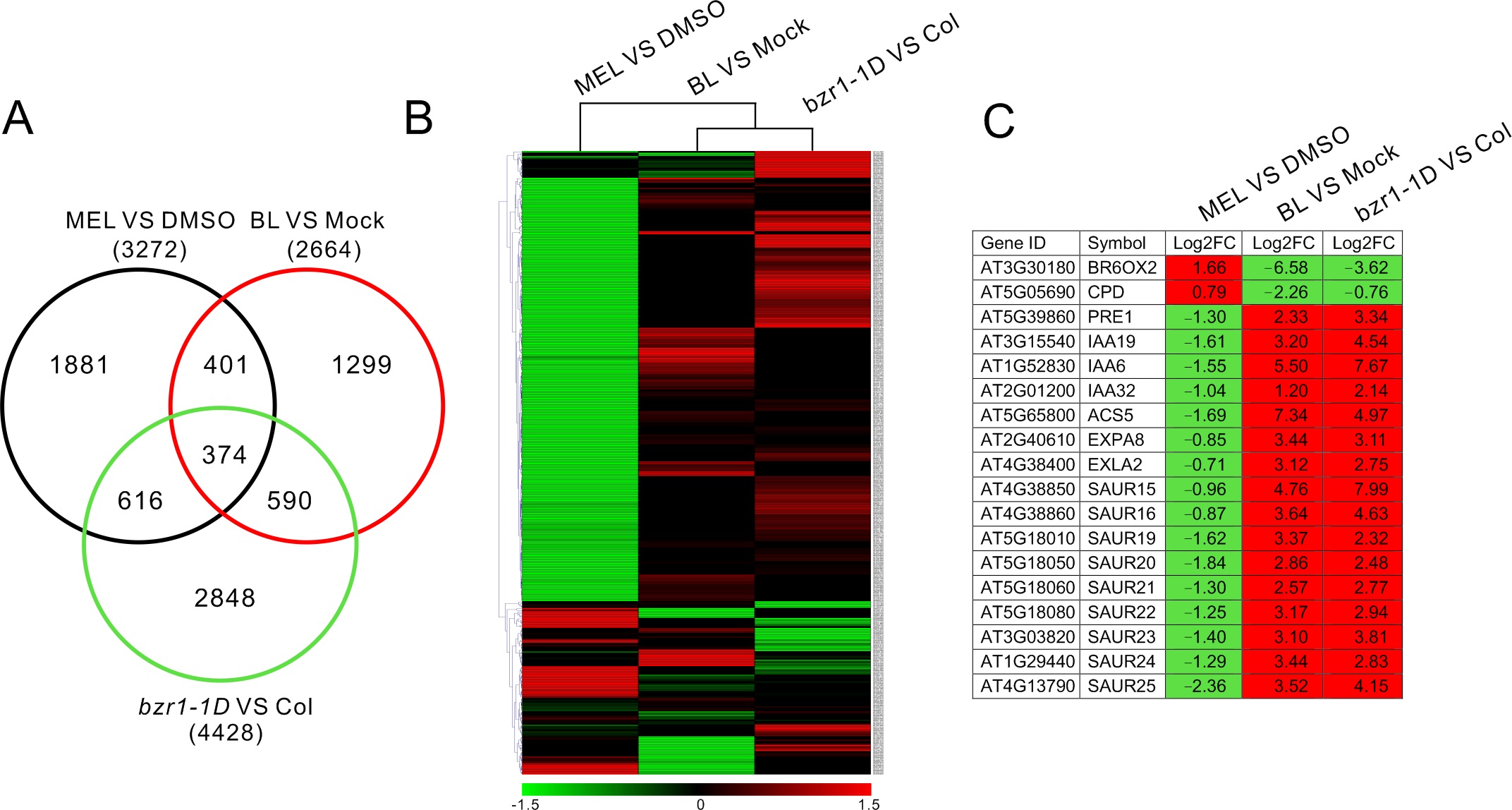

Supplement: Supplementary Figure S1 — Exogenous melatonin inhibits seedling growth in Arabidopsis. [file DataSheet_1.zip › Supplementary files/Supplementary Figure S3 Comparative expression spectrum analysis.jpg]
